# Contactless recording of sleep apnea and periodic leg movements by nocturnal 3-D-video and subsequent visual perceptive computing

**DOI:** 10.1038/s41598-019-53050-3

**Published:** 2019-11-14

**Authors:** Christian Veauthier, Juliane Ryczewski, Sebastian Mansow-Model, Karen Otte, Bastian Kayser, Martin Glos, Christoph Schöbel, Friedemann Paul, Alexander U. Brandt, Thomas Penzel

**Affiliations:** 1 Charité – Universitätsmedizin Berlin, corporate member of Freie Universität Berlin, Humboldt-Universität zu Berlin, and Berlin Institute of Health, Interdisciplinary Center of Sleep Medicine, Berlin, Germany; 2 Department of Neurology, Bundeswehr-Krankenhaus, 10115 Berlin, Germany; 3 Motognosis GmbH, Berlin, Germany; 4 Charité – Universitätsmedizin Berlin, corporate member of Freie Universität Berlin, Humboldt-Universität zu Berlin, and Berlin Institute of Health, NeuroCure Clinical Research Center, Berlin, Germany; 5 Clinical and Experimental Multiple Sclerosis Research Center, Department of Neurology, Charité University Medicine Berlin, and Experimental and Clinical Research Center, Max Delbrück Center for Molecular Medicine and Charité University Medicine Berlin, Berlin, Germany; 6 Department of Neurology, University of California, Irvine, Irvine, CA United States; 7 International Clinical Research Center, St. Anne’s University Hospital Brno, Brno, Czech Republic

**Keywords:** Bioinformatics, Computational biophysics

## Abstract

Contactless measurements during the night by a 3-D-camera are less time-consuming in comparison to polysomnography because they do not require sophisticated wiring. However, it is not clear what might be the diagnostic benefit and accuracy of this technology. We investigated 59 persons simultaneously by polysomnography and 3-D-camera and visual perceptive computing (19 patients with restless legs syndrome (RLS), 21 patients with obstructive sleep apnea (OSA), and 19 healthy volunteers). There was a significant correlation between the apnea hypopnea index (AHI) measured by polysomnography and respiratory events measured with the 3-D-camera in OSA patients (r = 0.823; p < 0.001). The receiver operating characteristic curve yielded a sensitivity of 90% for OSA with a specificity of 71.4%. In RLS patients 72.8% of leg movements confirmed by polysomnography could be detected by 3-D-video and a significant moderate correlation was found between PLM measured by polysomnography and by the 3-D-camera (RLS: r = 0.654; p = 0.004). In total, 95.4% of the sleep epochs were correctly classified by the machine learning approach, but only 32.5% of awake epochs. Further studies should investigate, if this technique might be an alternative to home sleep testing in persons with an increased pre-test probability for OSA.

## Introduction

In medicine, contactless motion analysis using the Kinect 3-D-camera and subsequent VPC is an effective diagnostic instrument for measuring gait impairment in patients suffering from multiple sclerosis (gait speed, stride length, stride time), tremor in Parkinson patients, fall risk assessment of elders, gesture commands, body sway and posture, anthropomorphic measurements for example for surgical planning, epileptic seizure semiology and even contactless heart rate measurement^1–15^. The Microsoft Kinect includes different sensors. At first, it includes a high definition 30 Hertz camera, similar to most of modern webcams and a microphone array. Moreover, the Kinect emits infrared light from three internal sources. This light is reflected by the environment and captured by an infrared sensor. Based on these infrared data, it can be calculated for each pixel, how long the light needs to be sent and captured again, and a 3-D-image of the environment can be built. Although the technical characteristics are ideal to examine sleep disorders, only few studies used the Kinect during the night. One study has monitored the sleep position of elder persons in the bed during the night by VPC in order to investigate the risk of falling^16^. Krüger *et al*. investigated one person, Procházka *et al*. four persons, and Garn *et al*. 10 persons simultaneously with the Kinect and polysomnography (PSG)^17–19^. Procházka *et al*. found a very good correlation of breathing features, and Garn *et al*. a good correlation of periodic limb movements (PLM)^18,19^.

Vision-based and hence contactless movement analysis using a 3-D-camera was originally developed for game consoles. However, the use of 3-D-cameras is not limited to the entertaining industry; they have been employed also with promising results in medicine^1–3^. 3-D-cameras have been originally developed by Microsoft, who indeed discontinued the production of the so called “Kinect” sensor and adapter, but continued to provide a free Software Development Kit (SDK) (https://developer.microsoft.com/en-us/windows/kinect). Almost all study groups using 3-D-cameras for motion analysis are using the Microsoft Kinect and SDK for 3D video capture^16^. Subsequently, after recording and after signal extraction computational algorithms are needed to interpret the flood of data. Therefore, most study groups program their own visual perceptive computing (VPC) software tools.

The aim of this study was to investigate whether VPC could *i*) be a home-based measurement for obstructive sleep apnea (OSA) comparable to home sleep testing (HST), *ii*) detect PLM, and *iii*) differentiate between sleep and wake.

OSA and RLS patients aged 18 to 70 years were consecutively recruited between September 23, 2015 and November 20, 2016 from our outpatient sleep center and healthy volunteers through personal contact. All OSA patients were newly diagnosed and untreated and underwent a nocturnal HST prior to enrollment. Complete data sets of simultaneously recorded PSG and Kinect measurements were obtained from 59 persons: in the OSA subgroup 11 men and 10 women aged 27 to 64, in the RLS subgroup 10 men and 9 women aged 20 to 70, and in the healthy volunteer (HV) subgroup 9 men and 10 women aged 18 to 57.

## Results

### Apnea hypopnea index in the polysomnography versus respiratory events measured with the Kinect

**Figure 1:**
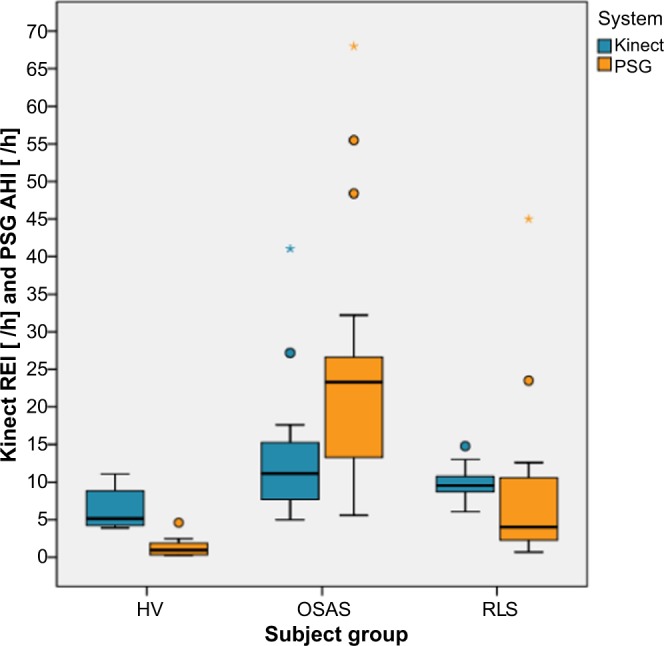
Box-Whisker-Plot comparing respiratory events measured with the Kinect versus apneas and hypopneas measured with polysomnography. In healthy volunteers and RLS patients, the Kinect overestimates respiratory events and in OSA patients the opposite is the case. Abbreviation: AHI = apnea/hypopnea index; HV = healthy volunteer; Kinect REI = Kinect respiratory event index; PSG = polysomnography; OSAS = obstructive sleep apnea syndrome; RLS = restless legs syndrome.

Kinect-respiratory-event-index *(KREI) versus apnea-hypopnea-index (AHI):* Fig. 1 shows a Box-Whisker-Plot comparing respiratory events measured with the Kinect (KREI) versus apneas and hypopneas measured with polysomnography (AHI). It indicates the variability outside the upper and lower quartiles in the different subgroups.

**Figure 2:**
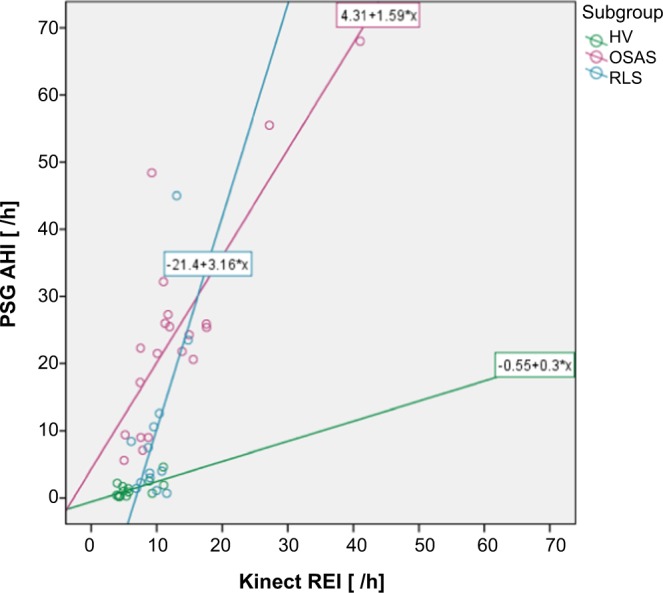
Linear relationship between respiratory events measured with the Kinect and the polysomnography. There is a linear relationship between respiratory events measured with the Kinect and measured with the polysomnography. Abbreviation: AHI = apnea/hypopnea index; HV = healthy volunteer; Kinect REI = Kinect respiratory event index; PSG = polysomnography; OSAS = obstructive sleep apnea syndrome; RLS = restless legs syndrome.

In healthy volunteers and RLS patients, the measurement with the Kinect overestimates respiratory events and in OSA patients the opposite is the case. (Ten RLS patients suffered from comorbid OSA, for which reason the AHI is also increased in this subgroup). Moreover, there is a linear relationship between respiratory events measured with the Kinect and the PSG which is shown in Fig. 2.

In the OSA subgroup there was a highly significant and strong correlation between the AHI and the KREI (r = 0.823; p < 0.001), and a significant moderate correlation in HV (r = 0.647; p = 0.013) and in RLS patients (r = 0.616; p = 0.025).

*KREI*_*SLEEP*_ *versus AHI:* Analysing the relationship between the AHI and KREI_sleep_ there was quite similar a highly significant and strong correlation in the OSA subgroup (r = 0.826; p < 0.001), and a significant moderate to strong correlation in HV (r = 0.666; p = 0.009); whereas in RLS there was only a trend (r = 0.530; p = 0.063).

*KREI versus AHI*_*obs*_: After adjusting for central apneas and analyzing exclusively respiratory events with continued breathing efforts (obstructive sleep apnea) similar results were found. In the OSA subgroup there was a similarly significant correlation between the AHI_obs_ and the KREI (*r* = 0.887; p < 0.001), a significant moderate correlation in HV (*r* = 0.701; p = 0.005), but no correlation in RLS patients (r = 0.445; p = 0.128).

**Figure 3:**
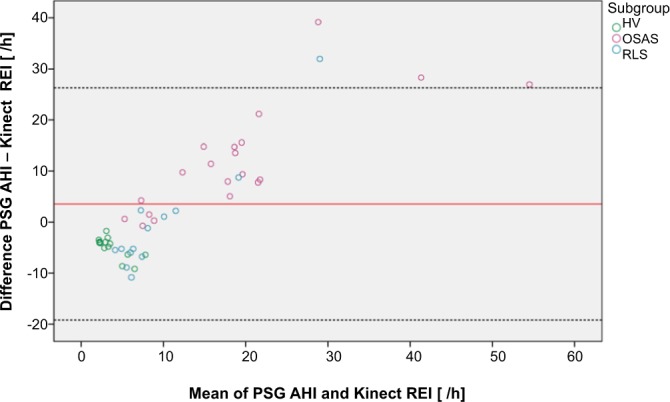
Respiratory events respectively AHI measured with the Kinect respectively PSG. The Bland–Altman plot comparing apneas/hypopneas measured by polysomnography (AHI) and by the Kinect (KREI) showed a systematic difference (mean difference 3.6; p = 0.041; see Fig. 3), which is negative for healthy volunteers - meaning that the AHI was lower in this subgroup compared to the KREI. In patients suffering from OSA the difference was positive, which implies that the AHI was higher compared to the KREI in this subgroup. Abbreviation: AHI = apnea/hypopnea index; HV = healthy volunteer; Kinect REI = Kinect respiratory event index; PSG = polysomnography; OSAS = obstructive sleep apnea syndrome; RLS = restless legs syndrome.

The Bland–Altman plot in Fig. 3 below shows, that measurements with the Kinect tend to underestimate respiratory events in OSA patients.

To test the validity of the Kinect as a diagnostic device a receiver operating characteristic (ROC) curve was generated. It shows, comparing the OSA subgroup with the HV subgroup, a sensitivity of 90% for detection of OSA and a specificity of 71.4% - applying a KREI cut-off of 6.6 per hour (area under the curve 0.857).

In order to investigate whether the difference between the KREI and the AHI is caused by the sensitivity of the Kinect sensor (modifications of the breathing signal) or the computing algorithm, we performed retrospectively a simultaneous visual scoring of the row data of the Kinect and the PSG. By visual scoring, only 77% of the apneas and hypopneas from the PSG could be seen in the Kinect data which suggests that the problem has not been caused by the computing algorithm, but rather by the sensor itself and it is unlikely that optimization of VPC will improve the results.

### Periodic limb movement index in the polysomnography versus Kinect measurements

By visual scoring of the raw data of the leg movements (Kinect versus PSG), 72.8% of leg movements in the PSG could be seen in the Kinect data. In HV and RLS patients there was a significant correlation between the PLMI and the KPLMI (RLS: *r* = 0.654; p = 0.004/HV: *r* = 0.686; p = 0.002), whereas no correlation was found in OSA patients (*r* = 0.323; p = 0.207).

### Sleep detection by visual perceptive computing and subsequent machine learning

The aim of the machine learning concept was to develop algorithms which can automatically detect sleep based on a motion analysis – independent from the electroencephalography. For technical reasons the Kinect data were stored in different sections of two hours and in most cases a single night included four parts of Kinect recordings. For the following machine learning approach, only artefact free parts were used.

The Kinect data of the first 12 participants were segregated in analogy to the PSG data into 30 second epochs and we performed a motion analysis of the different polysomnographically confirmed sleep stages. Subsequently, we developed an algorithm of sleep stage classification based solely on the Kinect data, what means that these new machine learning algorithms can classify sleep only by an analysis of movements (Kinect sleep stages) without needing any PSG data. The data of the first 12 participants served exclusively as a training set and were not examined further.

In a second step, we used then these new algorithms to predict sleep in the remaining participants whose data have not been used for the development of the above-mentioned new algorithms.

In a third step, the obtained Kinect sleep stages were compared to the PSG sleep stages. In total, 95.4% of the PSG sleep epochs were correctly classified by the machine learning approach, but only 32.5% of awake states. This results in a positive predictive value of sleep detection of 77.9% and a negative predictive value of sleep detection of 74.1%.

In particular in the sleep onset period (calm awakening) when participants were lying in bed without moving and tried to fall asleep, the machine learning algorithm classified often wrongly sleep instead of awake – probably due to the lack of movements in this period.

## Discussion

This is the largest prospective study using VPC to measure respiratory events, PLM and sleep stage classification based on a motion analysis during the night. If applying a cut-off of 6.6 respiratory events per hour (KREI), the Kinect discovered OSA with a sensitivity of 90% and a specificity of 71.4%. These findings suggest that 3-D-video could perhaps become an alternative to HST.

Reichert *et al*. investigated 51 consecutive adults referred to the sleep lab for suspicion of OSA, and they found OSA using HST in 91% and specificity was 83%^20^. Against this background it is especially notable that in our study only approximately one-third of persons had an increased pre-test probability for OSA - in contrast to most studies investigating the sensitivity of HST, mostly predominantly in patients suspected of having OSA^21,22^. The scoring of hypopneas requires according to the AASM rules the measurement of flow and arousal and oxygen saturation^23^. Although 3-D-video measures neither arterial oxygen saturation nor arousal or flow, the Kinect can help probably in a comprehensive evaluation for sleep disordered breathing. It seems to detect apnea and hypopnea with a sensitivity and specificity comparable to that of three channel HST. (*It should be noted that HST measures flow and oxygen saturation*, *but nevertheless does not measure arousal which is why arousal-related hypopneas (type B) are consequently overlooked in HST; arousal can only be detected by EEG and the measurement of arousal requires a PSG*. *Hence*, *both methods*, *3-D-video and HST cannot detect arousal-related hypopneas*). This might partially explain the differences between the AHI measured with the Kinect and the PSG.

The diagnosis of OSA requires a clinical evaluation and an assessment of breathing patterns. Both, HST and 3-D-video measurements can fail to identify mild OSA which may result in misdiagnosis and lack of therapy^24^. Therefore, OSA can only be excluded by PSG and not by HST. In case of suspicion of OSA a PSG is in most cases required in order not to misdiagnose mild OSA - even if a prior HST has been normal. This is important in order to describe the limitations of the HST. Measurements with 3-D-video seem to detect apnea and hypopnea with a sensitivity and specificity comparable to that of three channel HST.

It should be noted that the Kinect system includes a microphone array. An audio analysis of snoring might improve additionally diagnostic accuracy. However, in this study we did not investigate the combined parameter of simultaneous changes in breathing amplitude *and* snoring; but this should be done in further studies.

The Portable Monitoring Task Force of the AASM recommended for the diagnosis of OSA home diagnosis with portable monitoring devices in conjunction with a comprehensive sleep evaluation by a sleep specialist as an alternative to PSG in patients with a high pretest probability of moderate to severe OSA and without significant comorbid medical conditions or other sleep disorders^24^. Our results indicate that the Kinect may be appropriate for these purposes and may become a new diagnostic tool in timely home-based diagnosis of OSA and for therapy control in patients under CPAP therapy. The results do not depend on the sleep stage classification. Even if analyzing only the time spent sleeping (KREI_sleep_) the results did not change significantly.

This is especially important considering the background that in our study the sleep stage classification based on the motion analysis was not successful. Krüger *et al*. and Procházka *et al*. found a good correlation of the sleep stage classification by the Kinect compared to PSG^17,18^. But it should be noted, that Krüger *et al*. investigated only one person, and Procházka *et al*. investigated (apart from the small sample size with four persons) breathing patterns for the sleep stage classification and that in our study approximately the half of the patients suffered from OSA (21 OSA patients and 10 RLS patients with comorbid OSA).

There are several possibilities to explain why the motion analysis was not successful. One the one hand, it must be said that for that purpose larger samples are required and our study should be seen as a preliminary study. It may well be the case that further studies will achieve the objective to classify sleep based on movements during sleep. Moreover, as a further methodical limitation, the algorithm could not be accurate enough. All in all, it could be that a machine learning approach with a bigger sample size could be able to classify sleep only by a motion analysis. On the other hand, it is possible, that a sleep stage classification by motion analysis might be generally not successful. Up to date, very few data exist on the frequency and number of movements in different sleep stages^25,26^. Stefani *et al*. investigated movements during physiological sleep in the head, neck, trunk, and in the extremities in a single night PSG^25^. They found a median number of 10.2 movements per hour sleep, most frequent in N1 and to a lesser extent in REM, and lowest in N2 and N3. But these results can be influenced by the so-called first-night-effect due to cables and the unusual environment. Moreover, to our knowledge, there are no valid data about the amount of physiological movements during the time awake before sleeping and during nocturnal awake phases. This situation is complicated by the fact, that in patients suffering from RLS and in persons with periodic limb movements in sleep (PLMD) the beginning of sleep is not necessarily associated with a decrease of movements. Finally, we do not have normative data about the amount of movements during the bed-time in healthy subjects (awake and asleep). The situation appears even more complicated if we consider, that in OSA patients movement arousal due to desaturation can be associated with or without sleep stage changes or awakenings. Finally, a sleep stage classification by a 3-D-video-camera and subsequent motion analysis uses the parameter “motion” for the sleep stages classification and for long there has been a debate, if actigraphies are able to detect sleep and in which extent. Our approach to classify sleep by a motion analysis does not substantially differ from previous studies using actigraphy. The only difference is that we used a 3-D-video for motion caption, but the principles are the same. Previous sleep studies using actigraphy showed indeed only a moderate correlation between the TST measured concurrently with PSG and actigraphy, due to an overestimation of TST by actigraphy^27^. Moreover, several studies suggest some systematic misclassification of sleep and wake by actigraphy^27^. It must be highlighted, that our machine learning approach classified sleep correctly but misclassified the calmer wake periods during the phase before falling asleep as sleep - and these calmer periods before falling asleep were not distinguishably from wake, because patients did not move during this period. This is in line with previous studies showing, that actigraphy seems to underestimate sleep onset latency (SOL) and wakefulness after sleep onset (WASO)^28^. However, previous studies using actigraphy showed that actigraphy is appropriate for the assessment of and stability of treatment effects of anything from hypnotic drugs to light treatment to continuous positive airway pressure^29^. Against this background, it remains to be analyzed whether measurements with 3-D-video might also be suitable to assess treatment effects. Actigraphy has also been shown to be very good for identifying rhythms^29^. Here, too, it is conceivable that 3-D-video measurements may be suitable for the contactless investigations of rhythms (which should be investigated by further studies).

The Kinect detected approximately three-fourths of PLM measured with the PSG. This might be due to the fact, that a certain number of PLM measured by bipolar EMG are not accompanied by a visible movement. On the other hand, the Kinect can detect movements from the whole body. PLM are mostly caused by a periodic innervation of the tibialis anterior muscle with subsequent extension of the big toe and often with a flexion in the knee and sometimes also in the hip^30^. Apart from the chin EMG, the AASM 2012 rules recommend solely the EMG of both tibialis anterior for detection of movement^23^. Apart from some sleep laboratories with particular expertise in REM sleep behavior disorder and other movement disorders, the routinely done standard video-PSG contains only the EMG of both tibialis anterior for detection of motor activity of the limbs and trunk. However, the sleep specialist can use the video in order to analyze movements what is very time-consuming and not used on a regular basis.

We have conversely not explored how many movements are visible in the Kinect, but not in the tibial EMG. Provini *et al*. investigated PLM in detail by PSG and extended surface EMG (biceps brachii, triceps brachii, rectus abdominis, thoracolumbar paraspinales, rectus femoris, biceps femoris, tibialis anterior and gastrocnemius muscles). Nearly three-fourths of PLM involved the tibialis anterior, but PLM also involved the gastrocnemius (antagonist of the tibialis anterior), biceps femoris, rectus femoris and even in axial muscles (rectus abdominis and thoracolumbar paraspinales)^31^. This is not surprising, given that the RLS is a sensimotor disorder with an altered dopaminergic neurotransmission in the brain with activation of the ascending arousal system and loss of the inhibitory influence on the spinal cord^32–34^.

Even if the exact mechanism of idiopathic RLS remains unclear, low brain iron despite normal peripheral iron due to a decreased iron acqusition by brain cells in genetically predisposed persons is one of its key findings^35^. The brain iron insufficiency is associated with an altered dopaminergic neurotransmission which is considered the final mechanism underlying restlessness and PLM^32^. Autopsy data showed a decreased brain iron in the substantia nigra and putamen with an increased activity of tyrosin hydroxylase (tyrosine hydroxylase is catalyzing L-tyrosine into levodopa)^32,36^. All in all, brain iron insufficiency seams to lead to an increased dopamine synthesis. Therefore, it is not surprising, that PLM involve more muscles than just the tibialis anterior and more EMG should be recorded. But this is often not possible because many PSG systems have not enough free channels in order to record more EMG and only sleep laboratories with special expertise in REM sleep behavior and movement disorders are recording EMG from all four limbs and from the trunk.

Provini *et al*. investigated the time delay and pattern of activation between the first and the other activated muscles. The tibialis anterior was the most frequent starting muscle with approximately the half of PLM starting in the tibialis anterior^31^. However, some PLM started also in the gastrocnemius, biceps or rectus femoris, in the upper limb muscles and 1.1 per cent even in axial muscles (rectus abdominisa and thoracolumbar paraspinales). Moreover, detailed analysis of the pattern of activation showed no constant recruitment pattern from one to another PLM, even in the same patient and the time delay between the first and the last activated muscles was up to two seconds. A major advantage of the 3-D-video is, that it is contactless and only a minimum of time is required for starting and stopping of the records so that movements from the whole body can be investigated and also repeated investigations can be performed with a minimal investment of time. Further investigations of other sleep disorders as for example REM sleep behavior should be performed.

In sum, the Kinect seems to be a valuable device for home-based measurements and might be an alternative to home sleep testing in patients with a high pretest probability of moderate to severe OSA and without significant comorbid medical conditions or other sleep disorders. However, future prospective and controlled studies are required to examine whether this new technology is a suitable diagnostic tool for home sleep testing at home.

Moreover, this new VPC-based technology has the potential to become also a valuable diagnostic instrument to measure PLM and other movements during sleep. Considering the background of current technical developments and a shift toward home-based investigations, normative values about the frequency and nature of movements during the bedtime are recommended and future studies should be performed.

## Method

### Subjects

**Figure 4:**
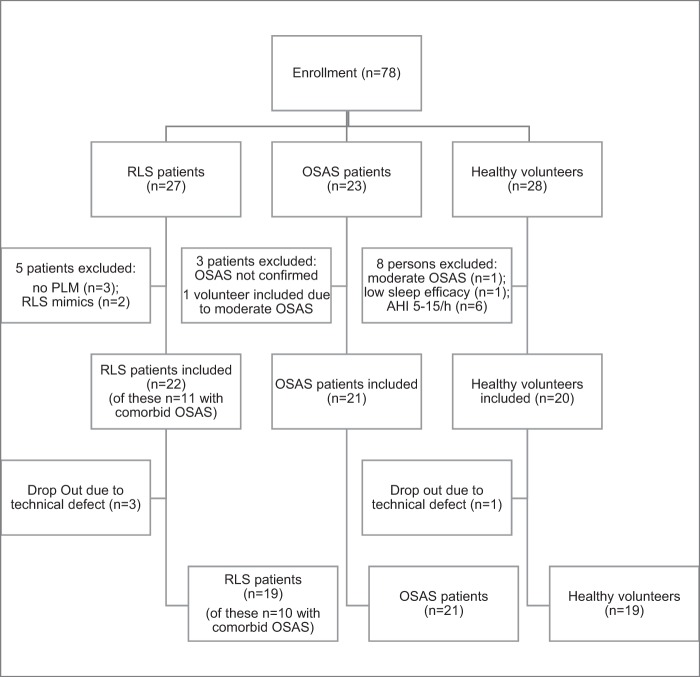
Consort flow diagram. From the enrolled 23 OSA patients, OSA was confirmed in 20 patients, and one person with moderate OSA from the healthy volunteers entered in this subgroup; therefore, 21 OSA patients were finally included. In the RLS subgroup, three RLS patients did not show PLM in the PSG and in two cases the diagnosis was changed into RLS mimic. Subsequently, these five patients were excluded. In sum, 63 persons were included in this study. Due to technical deficits (recording, headbox, synchronization, storing) four persons were lost. Complete data sets of simultaneously recorded PSG and Kinect measurements were obtained from 59 persons: in the OSA subgroup 11 men and 10 women aged 27 to 64, in the RLS subgroup 10 men and 9 women aged 20 to 70, and in the HV subgroup 9 men and 10 women aged 18 to 57. Abbreviation: AHI = apnea/hypopnea index; OSAS = obstructive sleep apnea syndrome; RLS = restless legs syndrome.

Figure 4 displays the sampling process. OSA and RLS patients aged 18 to 70 years were consecutively recruited between September 23, 2015 and November 20, 2016 from our outpatient sleep center and healthy volunteers through personal contact. All OSA patients were newly diagnosed and untreated and underwent a nocturnal home sleep testing prior to enrollment.

The OSA diagnosis was based on the AASM criteria and we used the cutoffs for OSA severity described there (AHI: 5–15 mild, 15–30 moderate, and >30 severe)^37^. If OSA was not confirmed by the subsequent performed in-laboratory PSG, patients were not included. All RLS patients were consecutively included and fulfilled the four essential RLS criteria^38,39^.

Twenty-three OSA patients, 27 RLS patients and 28 healthy volunteers were enrolled. From the 28 healthy volunteers 7 showed an increased apnea hypopnea index (AHI) ≥ 5/h (of these, one patient showed moderate OSA with an AHI of 15/h who was subsequently included in the OSA subgroup) and one patient a low sleep efficacy. Therefore, 20 healthy volunteers were included in this subgroup.

All participants with a known or suspected diagnosis or history of heart failure, home ventilation, continuous positive airway pressure (CPAP) therapy, respiratory insufficiency, mental disorder or class III obesity were excluded. Moreover, pregnancy was an exclusion criterion. By definition, all OSA patients showed apneas or hypopneas.

Drugs were not systematically tapered in RLS patients, but in some patients, dopamine agonists were reduced the evening before the examination. The absence of PLM in the PSG was considered as an exclusion criterion for the RLS subgroup, since the main interest in the RLS subgroup was the analysis of PLM.

Ethics statement. Permission for the study was obtained from the Charité University Medicine Berlin Ethics Committee on 21 September 2015 (EA1/199/15). All methods were carried out in accordance with the relevant guidelines and regulations. All patients gave prior written informed consent.

Clinical trial. Clinical Trial Name: Pilot study on contactless sleep monitoring of patients with different sleep disorders based on three-dimensional video signal and audio signal analysis (“Kinect”); http://www.germanctr.de; DRKS-ID: DRKS00009347; registration date: 27/11/2015.

### Procedures

Polysomnography. The PSG with audio-video recording were performed and scored with the Embla system (N7000 or S7000; Natus; Software Remlogic 3.4, Embla Systems, Ontario, Canada) according to the AASM Manual for the Scoring of Sleep and Associated Events^23^. We used respiratory inductive plethysmography and nasal pressure transducer^23^. Sleep stages were scored manually according to the AASM Manual by the same board-certified sleep specialist (B.D.)^23^. Subsequently, the AHI was calculated (AHI).

Whereas obstructive apneas are associated with continued or even increased respiratory effort, central apneas are associated with an absent inspiratory effort. Based on these considerations, and moreover taking the fact into account that the Kinect is not measuring the flow, it would be possible that obstructive apneas with continued respiratory effort will be overviewed by the Kinect which might detect mostly central apneas, so that a shift to central apneas will occur. After an exploratory analysis during the first measurements, we found that this was not the case and that even a minor modification of the breathing amplitude was enough to detect an obstructive apnea by the Kinect. However, in order to evaluate the diagnostic accuracy of the detection of OSA and to better evaluate and compare the two methods (PSG and Kinect) we created in parallel an AHI_obs_ containing only obstructive and mixed apneas and hypopneas.

The bipolar electromyogram (EMG) from the left and right anterior tibial muscle was used to record leg movements. The AASM Manual defines a leg movement as a period of 0.5–10 seconds with an increase in EMG voltage of 8 μV above resting EMG. PLM are defined as the consecutive sequence of at least four leg movements with intervals between 5 and 90 seconds. A PLM index (PLMI) was calculated.

3-D-video (Kinect). The patients were sleeping under a normal blanket. This means, that the 3-D-video captured mostly in the strict sense of the term the movement of the blanket. We used a Motognosis Labs System V1.0 (Motognosis, Berlin, Germany) equipped with a Kinect V2 for Windows sensor (9.8 × 2.6 × 2.4 inchs) and the Kinect Software Development Kit (SDK) version 2.0 (Microsoft, Redmond, WA, USA). The sensor and software was previously validated against a marker based system for medical motion analysis^40^. The software was adapted as recording software based on the programming language C#. We implemented a recording software based on the programming language C#. The recorded 3-D-video (30 frames per second) was digitized in the Microsoft XEF format and then processed with Matlab R2015b (MathWorks Inc., Natick, Massachusetts, United States).

**Figure 5:**
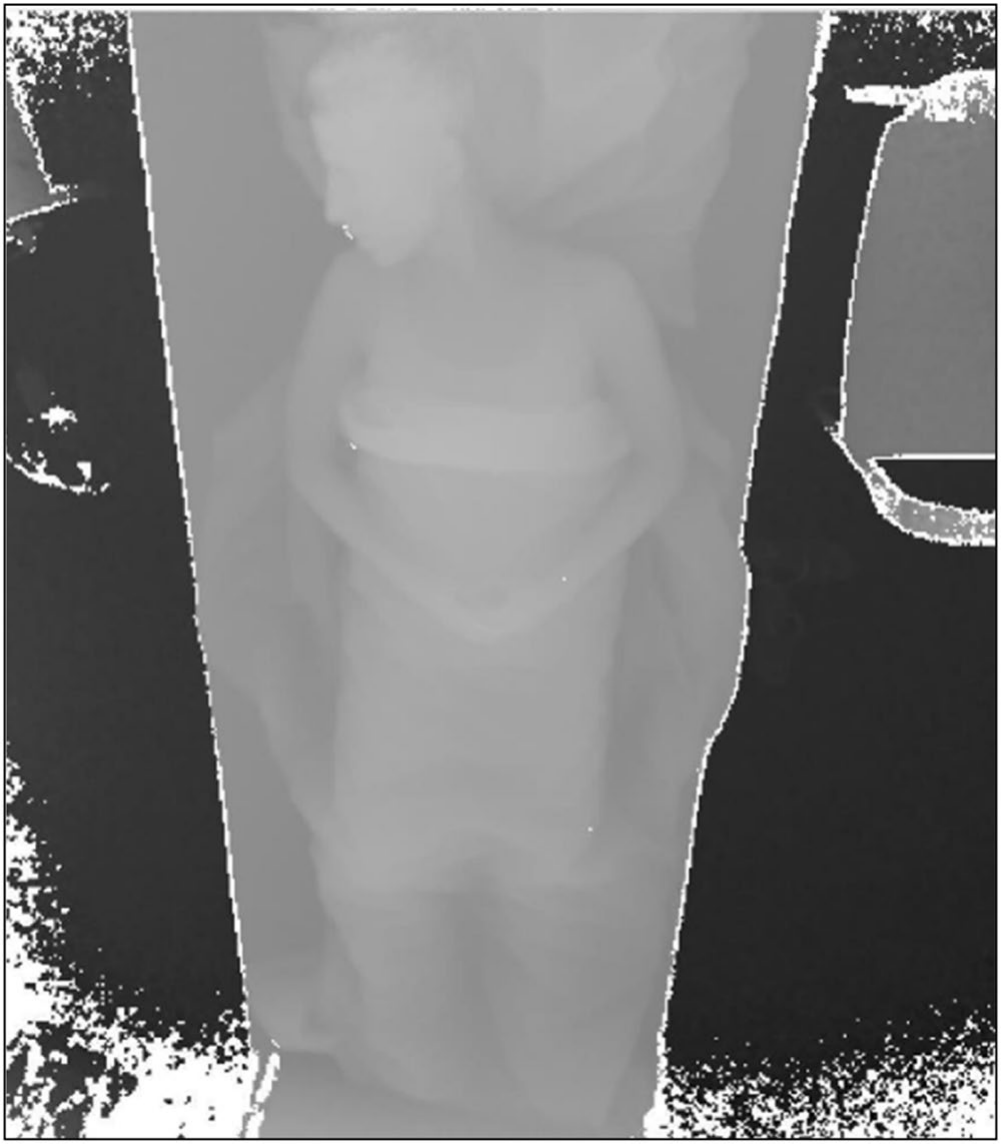
Generation of the depth image from the point cloud. The three-dimensional point clouds were rotated to remove perspective distortion.

We generated three-dimensional point clouds (see Fig. 5) which were rotated to remove perspective distortion and we employed several filters (flood-fill-operation, average-filter). After segmentation into a thoracic and a lower body segment in a multi-step procedure a circular section was placed on the thorax for the extraction of the breathing signal. The oscillating movements in this area reflect the respiratory effort.

For the signal of leg movements, a vertical was drawn from head to toe. All pixels below the vertical surface in the area of the legs form the signal for leg movements. As the Kinect measures neither the oxygen saturation nor the flow, only the changes of the respiratory effort, it cannot distinguish hypopneas from apneas.

Consequently, we calculated a Kinect-respiratory-event-index (KREI) including all hypopneas and apneas per hour from ‘lights off’ to ‘lights on’. Similarly, we calculated the Kinect-PLM-index per hour from ‘lights off’ to ‘lights on’ (KPLMI).

Both methods rely a variety of different thresholds for detection of respiratory and PLM events. The KREI is derived from the Kinect breathing signal by first calculating the signal envelope and afterwards using a sliding window approach to recognize local drops in the breathing amplitude. These drops are then classified as Kinect respiratory events if the drop in the amplitude is above a certain threshold over a certain period of time. Leg movements (also periodic leg movements) are derived from the Kinect movement signal by detecting peaks in the signal above a certain threshold. These peaks are then further filtered by various means (e.g. in close vicinity to whole body movements or according AASM close to a Kinect respiratory events). The values are oriented on the Embla parameters and fitted empirically on a subset of the data set and then kept fix for the remaining experiments. A Kinect PLM event is detected if enough remaining peaks fall into a long enough time interval. Figure 7 illustrates an example of the bipolar EMG on the right anterior tibial muscle and the Kinect leg movement detection.

**Figure 6:**
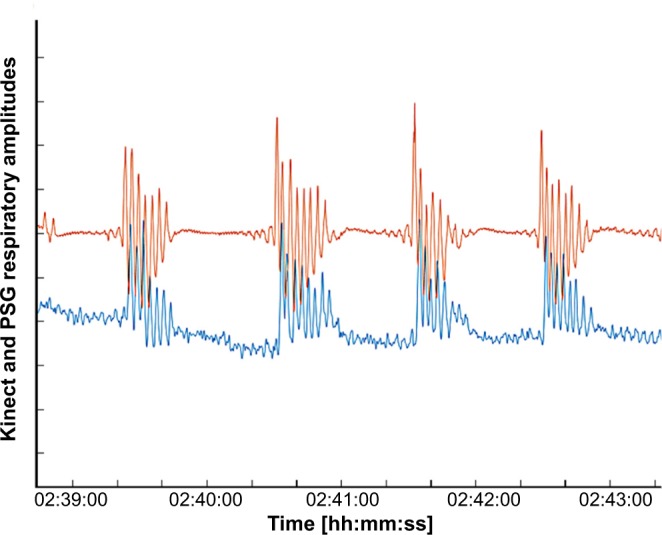
Comparison of the polysomnographical flow signal with Kinect breathing signal. Figure 6 illustrates an example of the Kinect breathing signal compared to the PSG flow signal. The flow signal is shown in orange; the Kinect breathing signal in blue. Abbreviation: PSG = polysomnography.

**Figure 7:**
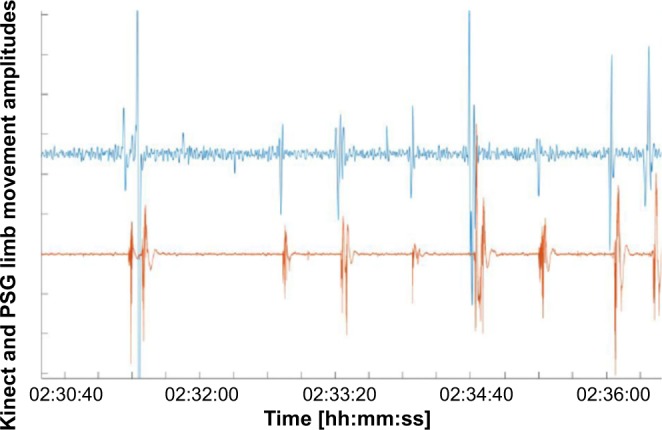
Comparison between the two-point electromyogram on the right anterior tibial muscle and the Kinect leg movement detection. We calculated the Kinect-PLM-index per hour from ‘lights off’ to ‘lights on’ (KPLMI). The polysomnographical signal of the right leg is shown in orange; the Kinect leg signal in blue. Abbreviation: PSG = polysomnography.

In addition, to better compare the two methods (PSG and Kinect), due to methodical reasons we created the KREI_SLEEP_ which represents all apneas und hypopneas per hour of sleep. For this purpose, we added the information about sleep stage classification from the Embla system into the Kinect data. Similarly, we calculated the KPLMI_SLEEP_ which contains all PLM per hour of sleep measured with the Kinect. Regarding the sleep wake discrimination based on the Kinect data, we used artefact free periods from 12 OSA patients for a machine learning approach and designed an optimized algorithm which has been used in the further course of the study for the sleep wake discrimination based on a motion analysis.

With regard to the distinction between sleep and wake, the machine learning approach was applied as previously described^17^. At first, the average change between two consecutive frames for an interval of 30 seconds were calculated (all in all 900 frames). These timeframes include the information about the sequence of a movement. Statistical parameters such as mean value, median, minimum and maximum were calculated. Additionally, the changes in the average pixel value over the time were analyzed – indicating the local manifestation of a movement. For these changes mean value, median, minimum and maximum were calculated as well. A raster is superimposed onto the mean change per pixel. For each field of the raster the mean value, median, minimum and maximum were calculated. We used an ensemble classifier of decision trees that was trained using RUSBoost to tackle the class imbalance between sleep and wake samples^41^. For training, the statistic parameters of the Kinect data and the PSG-hypnogram of 12 patients were used. In a next step the validity of the classification was examined.

### Statistical analysis

Following an exploratory analysis of the data we calculated the Pearson correlation coefficients to investigate the relationship between the AHI and the KREI (and between the AHI_obs_ and the KREI and between the AHI and the KREI_sleep_ respectively). Similarly, we investigated the relationship between the PLMI and the KPLMI. Bland–Altman plots were performed to evaluate the agreement between PSG and Kinect measurements. Statistical significance was established at p ≤ 0.05. Analysis was performed with SPSS software (IBM Corp. Released 2016. IBM SPSS Statistics for Windows, Version 24.0. Armonk, NY: IBM Corp).

## Data Availability

Raw data from the polysomnography study, protocols, and other metadata are available on request from the corresponding author (C.V.). Videos cannot be made publicly available due to ethic restrictions.

## Code availability

The code used in this study is available by contacting the corresponding author (C.V.).
